# The incidence and impact of anosmia on daily life after COVID-19 infection: A cross-sectional study in a tertiary center in Saudi Arabia

**DOI:** 10.15537/smj.2022.43.12.20220559

**Published:** 2022-12

**Authors:** Afnan F. Bukhari, Moayyad Malas, Kamal Hanbazazah, Faisal Zawawi

**Affiliations:** *From the Department of Otolaryngology-Head and Neck Surgery (Bukhari, Malas, Hanbazazah, Zawawi), King Abdulaziz University, and from the Department of Otolaryngology and Surgery (Malas), King Khaled Hospital, Ministry of National Guard, Jeddah, Kingdom of Saudi Arabia.*

**Keywords:** anosmia, quality of life, coronavirus, COVID-19, SARS-CoV-2

## Abstract

**Objectives:**

To investigate the impact of olfactory dysfunction’s (OD) on patients with coronavirus disease-19 (COVID-19) and evaluate the risk factors associated with it.

**Methods:**

This cross-sectional study analyzed patients who tested positive for COVID-19 over a period of 4 months (May–July 2020) and experienced OD and mild illness. Selected patients were given 2 scales Olfactory Disorders Negative Statement (QOD-NS) and Sino-nasal Outcome Test (SNOT-22).

**Results:**

A total of 256 patients were enrolled, out of which 196 had anosmia after COVID-19 infection. More than 75% of the participants were aged between 20-40 years and 64.3% were women. The mean score of the patients was 25.13 (SD 19.6) on the SNOT-22, while it was 4.9 (SD 4.85) on the QOD-NS. There was no association between anosmia and other comorbidities and factors (age, smoking history, allergic rhinitis history, asthma, and so on). Only 39% of patients who had anosmia after COVID-19 recovered in less than 4 months.

**Conclusion:**

Olfactory dysfunction is a common symptom of COVID-19 infection and it can take more than 4 months to recover. Nevertheless, this cohort reports a moderate impact on their quality of life due to anosmia.

**T**he novel coronavirus was first reported in Wuhan, China, before spreading rapidly worldwide at the end of December 2019. On the 11th of February 2020, the World Health Organization (WHO) officially labeled the illness as coronavirus disease 2019 (COVID-19). It is caused by the severe acute respiratory syndrome coronavirus 2 (SARS-CoV-2).^1,2^ As of April 2022, more than half a billion people got diagnosed with COVID-19, and over 6 million died. The SARS-CoV-2 is transmitted via direct contact and airborne transmission.^3^ The presentation of COVID-19 varies, and it can be mild, moderate, severe, or sometimes fatal. The most common symptoms are fever, sore throat, cough, headache, chills, loss of smell and taste, diarrhea, and dyspnea.^4,5^ Diagnosis of COVID-19 is based on virological detection by polymerase chain reaction reverse transcription (PCR-RT) testing of the nasal cavity, nasopharynxl or throat swabs.^3^ Olfactory dysfunction (OD) commonly occurs in viral and post-viral upper respiratory tract infections, and the exact mechanism of action is not well understood. Multiple theories have been proposed to describe the pathophysiology of OD in COVID-19 patients. The 2 main theories for explaining OD in COVID-19 are mucosal edema with obstruction of airflow to the olfactory cleft, and post-viral neurodegeneration of the olfactory epithelium.^6^ Olfactory dysfunction is one of the most frequent symptoms of COVID-19, and patients with COVID-19 can present with OD as the only symptom.^7^ Olfaction plays an important role in daily living and affects food selection and enjoyment. Moreover, humans use their sense of smell to detect chemical and toxic hazards. Several studies have shown an important and strong relationship between OD and quality of life (QoL).^8^ Nevertheless, the literature is scarce when it come to the impact of anosmia after COVID-19 on daily life and its resolution. Although it is a common presentation of COVID-19, local and international research on this topic is scarce.

The main aim of this study is to investigate the impact of OD in patients with mild COVID-19 using patient-reported outcome questionnaires and to evaluate the risk factors associated with OD in COVID-19 patients.

## Methods

This cross-sectional questionnaire scale-based study, approved by our institutional ethics review board, and the study was carried out according to the principles of Helsinki declaration. The study focused on olfactory-related QoL and symptom burden in patients who had been infected with SARS-CoV-2.

The inclusion criteria were adult patients with COVID-19 infection, confirmed by a nasopharyngeal swab test and the presence of OD. The exclusion criteria were pediatric age group (<18 years), patients who had OD before acquiring COVID-19 infection, and severe infection requiring long duration of hospitalization or intensive care unit (ICU) admission. This study was carried out in a tertiary academic care hospital (Department of Otolaryngology-Head and Neck Surgery, King Abdulaziz University, Jeddah, Saudi Arabia). We used convenient selection randomization, in which 30% of the patients with positive COVID-19 tests were randomly contacted and enrolled in the study. Participants who met our criteria were contacted by phone and asked to fill out a questionnaire that consisted of demographic data and questions related to OD.

Additionally, patients were asked to complete 2 questionnaires: the Sinonasal Outcome Test (SNOT-22) and the Questionnaire of Olfactory Disorders Negative Statement (QOD-NS).

First, we used the SNOT-22 questionnaire, which has been validated in English and Arabic (locally native) languages.^9,10^ The score of this questionnaire is considered to reflect QoL, which is usually affected in patients with chronic rhinosinusitis and is used as an indication for surgery. The SNOT-22 has a score range of 0-110. For the second questionnaire, we used brief English and Arabic versions of the QOD-NS, which have been validated.^11,12^ A shortened version of this questionnaire was used (7 items) to improve the response rate and make it easier for participants to fill out. This questionnaire reflects the olfactory-specific dysfunction that occurs in patients with chronic rhinosinusitis. The scoring range for the QOD-NS is 0-21. In both of these questionnaires, a higher score indicates a worse QoL (Appendix 1-4??).

The participants’ were assigned anonymous numbers and coding for privacy and data were kept private and accessible only to the primary author. Means and standard deviations (SDs) were used for continuous variables, while frequencies and percentages were used for dichotomous variables. For comparative analysis, we used the Student’s t-test or ANOVA test, where applicable. Statistical significance was set at a *p*-value of <0.05. All analyses were performed using SPSS version 22 (IBMCorp, Armonk, NY, USA).

## Results

**Figure 1:**
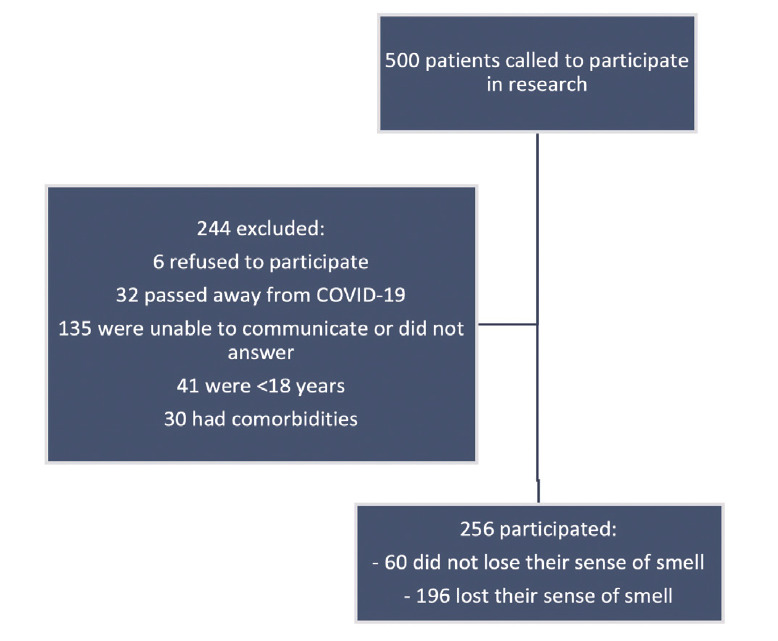
- A flowchart of the participants approach and selection.

We contacted 500 patients who were diagnosed with COVID-19 infection, of which 256 responded to the questionnaire (response rate, 51.2%) (Figure 1). After contacting our sampled population, we calculated the incidence of olfactory dysfunction to be 75.5%, where 60 responded that they did not have loss of smell and did not fill the questionnaire, and 5 answered the questionnaire that they did not experience olfactory dysfunction (60 of 256). The demographic characteristics of the participants are shown in Table 1. There was a female predominance (64.3%), and a majority of the participants was aged between 20 and 29 years. We excluded participants who complained of loss of smell prior to COVID-19 infection. Participants who did not experience any loss of smell (60 participants, 12.1%) were also excluded from the subanalysis. Almost half of the participants had experienced loss of sense of smell more than 4 months prior, as shown in Table 2. Additionally, some participants did not gain back their sense of smell or taste (14.8% and 11.2%, respectively).

**Table 1:** - Demographics details of the recruited subjects (N=196).

Characteristic	n	(%)
Gender (male)	70	(35.7)
***Age***		
Less than 20 years	17	(8.7)
20-29 years	100	(51.0)
30-39 years	51	(26.0)
40-49 years	20	(10.2)
50-59 year	6	(3.1)
60 and more	2	(1.0)
***Olfaction and gustation***		
Loss of smell	196	(100)
Loss of taste	162	(82.7)
Previous less of smell	0	(0)
***Comorbidities***		
Smoking	24	(12.2)
Asthma	31	(15.8)
Gastric reflux disease	14	(7.1)
Nasal allergy	21	(10.7)
Previous sinus surgery	8	(4.1)
Food or skin allergy	16	(8.2)
Chronic sinusitis	14	(7.1)
Hypothyroidism	5	(2.6)
Aspiring allergy	2	(1)
Obstructive sleep apnea	0	(0)
Medically free	96	(31.2)

**Table 2:** - Timing of diagnosis in relation to the timing of participating in the study.

Characteristic	n	(%)
Time of diagnosis of COVID-19		
During last week	10	5.1
1 week to 1 month ago	26	13.3
1 month to 2 months ago	22	11.2
		
2 months to 3 months ago	22	11.2
3 months to 4 months ago	22	11.2
More than 4 months ago	94	48
Persistence loss of smell	29	14.8
Persistence loss of taste	22	11.2

### Questionnaires to measure the effect of the loss of smell on daily activity

Participants completed 2 questionnaires to measure the effect of loss of smell on daily activities. A brief version of the QOD-NS (mean score of 4.96, SD 4.85). In the SNOT-22 test, (mean score was 25.13, SD=19.6). Additionally, we calculated the mean scores of each domain in the QOD-NS and found that the social and eating questions had the highest mean scores out of all domains, as shown in Table 3 However, a consensus on the interpretation of these tests is lacking. The low mean scores in this study suggest that hyposmia may have a mild-to-moderate effect on patients’ daily activities.

**Table 3:** - Questionnaire of Olfactory Disorders Negative Statement short version responses with mean and standard deviation (SD) for each item.

Subsections of the questionnaire	Totally disagree	Mostly disagree	Mostly agree	Totally agree	Mean±SD
***Social questions***					
The changes in my sense of smell make me feel isolated	(67.8)	(13.6)	(16.1)	(2.5)	1.93±2.32
Because of the changes in my sense of smell I have problems with taking part in activities of daily life	(66.8)	(16.1)	(14.1)	(3.0)	
Because of the changes in my sense of smell, I feel more anxious than I used to feel	(53.3)	(15.1)	(23.6)	(8.0)	
***Eating questions***					
Because of the changes in my sense of smell, I go to restaurants less often than I used to	(50.8)	(18.1)	(21.1)	(10.1)	1.95±1.94
Because of the changes in my sense of smell I eat less than I used to or more than I used to	(49.2)	(9.5)	(29.1)	(12.1)	
***Anxiety questions***					
Because of the changes in my sense of smell, I try harder to relax	(58.8)	(17.6)	(17.6)	(6.0)	0.71±0.77
***Annoyance questions***					
I am worried that I will never get used to the changes in my sense of smell	(52.3)	(16.1)	(24.6)	(7.0)	0.86±1.02
Total scores					4.9 (4.85)

Values are presented as percentage of a total of 196 participants.

### Duration of OD

Populations affected by COVID-19 show variable durations of persistent OD. Among our population, almost 85% of our population had regained their olfactory function at the time of survey (maximum of 8 months). Additionally, 40% of our sample regained their olfaction within 4 months after infection. However, 15% did not regain their olfaction by the time they answered the questionnaire, which ranged from 1-8 months after infection, as shown in Table 4.

**Table 4:** - Duration of olfactory dysfunction’s (OD) from COVID-19 infection (N=193).

Time of diagnosis	OD persist	OD recovered	Total OD recovery
During last week	0 (0)	3 (1.8)	3 (1.6)
1 week to 1 month ago	13 (44.8)	13 (7.9)	16 (8.3)
1 month to 2 months ago	2 (6.9)	22 (13.4)	38 (19.7)
2 months to 3 months ago	2 (6.9)	20 (12.2)	58 (30.1)
3 months to 4 months ago	5 (17.2)	18 (11.0)	76 (39.6)
More than 4 months ago	7 (24.1)	88 (53.7)	164 (85.0)
Total	29 (100)	164 (100)	193

Values are presented as number and percentage (%).

### Comparative analysis

We compared the mean test scores between variables such as age, gender, and the comorbidities described in Table 1. However, there were no significant differences in the mean test scores for any of the variables (*p*=0.245). On the other hand, although not statistically significant, we noticed an increase in the mean scores of the SNOT-22 test among the participants who were in the of 51-60 years age group (*p*=0.052), as well as a discrepancy in the scores among the different age groups in the QOD-NS test.

## Discussion

Despite its high prevalence in patients with COVID-19, OD did not have a severe effect on QoL. The SNOT-22 mean score of 25 obtained in our study suggests that OD has a mild-to-moderate effect on QoL. This score in patients with chronic rhinosinusitis (CRS) is usually not indicative of a major disease burden on patients.^13^

The incidence rate of OD was 25.3% in the COVID-19 population of our study. Variable incidence of OD has been reported in COVID-19 patients in the literature, with a range from 5.4% to 98.3%.^14^ Our results showed an average incidence rate consistent with that of previous studies. Moreover, the mean QOD-NS score in our study was 4.9, which reflects the mild effect of COVID-19-associated OD on the QoL of patients. In contrast, Elkholi et al^14^ studied the effect of OD in COVID-19 patients using the Multi-Clinic Smell and Taste Questionnaire and showed that in patients with OD due to COVID-19, there was a significant reduction in the QoL. Another study carried out in Saudi Arabia to evaluate the incidence and effect of OD in COVID-19 patients, using the brief QOD-NS test, revealed the incidence of OD to be 65.3%, with the most common symptom being headache (69%); OD was associated with a significant impact on the QoL of these patients.^15^ A similar study carried out in France by Vandersteen et al^16^ demonstrated a mean QOD-NS score of 11.1, indicating that OD has a modest effect on QoL.

The second objective of this research was to determine the possible associated risk factors with OD that decrease the QoL of COVID-19 patients. The majority of the patients included in this study were 20–40 years of age. This indicates a milder course of COVID-19 in this age group, resulting in a higher representation in our sample. We could not identify any significant risk factors associated with OD by comparing the mean scores for each comorbidity in our cohort. Notably, there was an increase in the mean scores of the SNOT-22 test among patients aged 51-60 years. However, this increase was not statistically significant when compared with the other age groups. These data are comparable to the risk factors for anosmia in patients with post-upper respiratory tract infection (URTI) and CRS. Olfactory dysfunction associated with URTI and CRS is more prevalent in middle-aged women (≥50 years), and these patients are at a higher risk of developing a more severe and permanent form of OD.^17-19^

The incidence of OD in COVID-19 patients appears to be comparable to that of CRS and URTI. Multiple studies have estimated the prevalence of OD caused by URTI to be as high as 20-40%.^21^ The duration of OD after URTI also remains variable. Rombaux et al^17^ found that 26% of the participants in their study demonstrated a significant improvement in their olfaction, but only 4% recovered to normosmia after 9 months. Similar research performed by Reden et al^18^ showed that 26% patients with OD related to URTI improved over an average period of 14 months. On the other hand, the prevalence of OD in patients with CRS varies among different studies, with the prevalence of anosmia as 18-38% and hyposmia reported 20-73%.^21,22^ Furthermore, the recovery from OD after CRS is related to the intervention type and duration of treatment.^19^

### Study limitation

First, the participants were mostly included after the resolution of their OD, and half of them had tested positive for COVID-19 more than 4 months before we contacted them. This may have caused them to answer that OD had a milder effect on completing the questionnaire. Second, we were not able to identify the risk factors for OD, as we only included patients with COVID-19 and OD in our analysis. Third, we included only patients with mild infections. Patients with moderate or severe COVID-19, who were not included in our study, may experience a higher impact of OD on their daily QoL. Fourth, we did not perform objective testing for olfaction or OD severity. The subjective perception of OD by the participants may have underestimated the actual incidence of milder OD, causing some cases to be excluded from our data. Fifth, our approximation of the duration of OD in our sampled population was not accurate. However, we can broadly see that the course of OD after COVID-19 lasts for less than four months in half of the population.

The small population of this study which could limit the interpretation of these results. Another limitation of this study is that not all patients had formal and objective olfactory assessment. Nevertheless, this study is the stepping stone to understanding further the olfactory dysfunction that follows COVID-19 infection and help physicians counsel patients on the possible resolution time of their anosmia. Additionally, future studies should focus on the difference in recovery rate between first wave of the COVID-19 pandemic and the other COVID-19 variants.

Olfactory dysfunction is one of the most frequent symptoms of COVID-19, and it has a moderate impact on patients’ QoL owing to its effect on daily activities. Our study results showed no identifiable risk factors associated with a higher impact of OD on the QoL of COVID-19 patients. However, female gender and advanced age, which are typically associated with the development of OD in patients with URTI and CRS, were significant factors in our population as well. Further studies are required to objectively assess OD in COVID-19 patients and to investigate the duration of OD and possible treatment protocols.

In conclusion, the OD is a frequently present symptom of COVID-19 infection. The majority of patients can take more than 4 months for them to recover their symptoms. Although anosmia in general affects the QoL of patients, this cohort described the anosmia’s impact to be less than severe.

## Figures and Tables

**Appendix 1:**
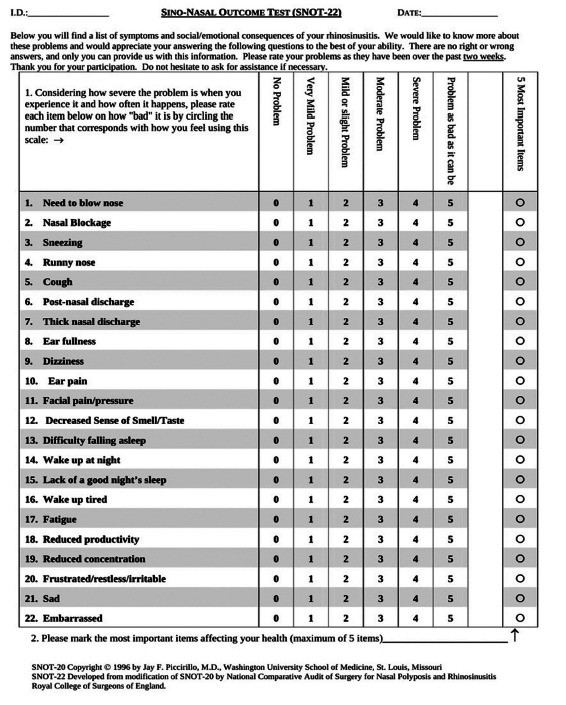
- The English version of Sinonasal Outcome Test questionnaire.

**Appendix 2:**
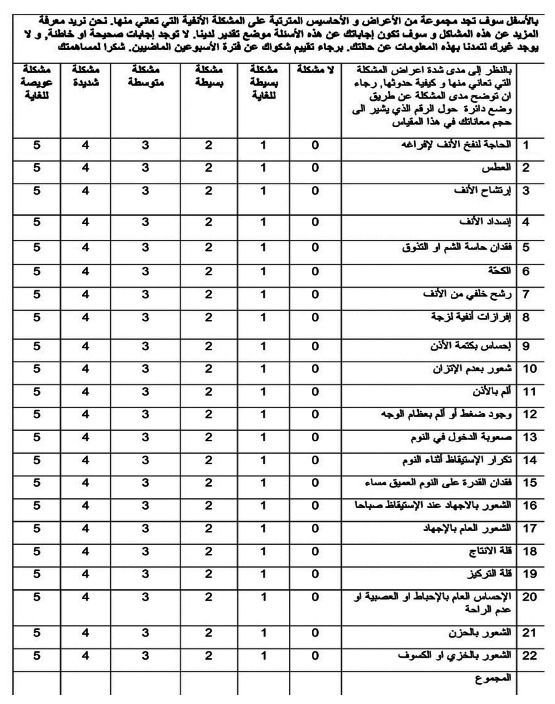
- The Arabic version of Sinonasal Outcome Test questionnaire.

**Appendix 4:**
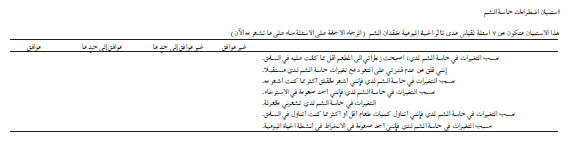
- The Arabic version of Sinonasal Outcome Test questionnaire.

## Appendix

**Appendix 3:** - Questionnaire of Olfactory Disorders Negative Statement. This questionnaire is based on 7 questions to measure the effect of decrease/loss of smell on daily activity, Kindly answer the questions based on what you feel right now.

Questions	Totally disagree %	Mostly disagree %	Mostly agree %	Totally agree %
***Social questions***				
The changes in my sense of smell make me feel isolated.				
Because of the changes in my sense of smell I have problems with taking part in activities of daily life.				
Because of the changes in my sense of smell, I feel more anxious than I used to feel.				
***Eating questions***				
Because of the changes in my sense of smell, I go to restaurants less often than I used to.				
Because of the changes in my sense of smell I eat less than I used to or more than I used to.				
***Anxiety questions***				
Because of the changes in my sense of smell, I try harder to relax.				
***Annoyance questions***				
I am worried that I will never get used to the changes in my sense of smell.				
